# Role of Antiplatelet Therapy in Patients with Severe Coronary Artery Disease Undergoing Coronary Artery Endarterectomy within Coronary Artery Bypass Surgery Ψ

**DOI:** 10.3390/jcdd10030112

**Published:** 2023-03-07

**Authors:** Ilir Balaj, Heinz Jakob, Ali Haddad, Fanar Mourad, Assad Haneya, Ebrahim Ali, Noura Ryadi, Matthias Thielmann, Arjang Ruhparwar, Sharaf-Eldin Shehada

**Affiliations:** 1 Department of Thoracic and Cardiovascular Surgery, West German Heart and Vascular Centre, University Hospital Essen, University Duisburg-Essen, 45147 Essen, Germany; 2 Department of Anaesthesiology and Intensive Care Medicine, University Hospital Essen, University Duisburg-Essen, 45147 Essen, Germany; 3 Department of Cardiac and Vascular Surgery, Campus Kiel, University-Medical-Center Schleswig-Holstein, 24106 Kiel, Germany

**Keywords:** end-stage coronary artery disease, viable myocardium, coronary artery endarterectomy, mono and dual antiplatelet therapy

## Abstract

*Background*—Coronary endarterectomy (CEA) has been introduced to allow revascularization in end-stage coronary artery disease (CAD). After CEA, the injured remnants of the vessel’s media could result in fast neo intimal tissue ingrowth, which require an anti-proliferation agent (antiplatelet therapy (APT). We aimed to review outcomes of patients undergoing CEA within bypass surgery who received either single-APT (SAPT) or dual-APT (DAPT). *Methods*—We retrospectively evaluated 353 consecutive patients undergoing CEA within isolated coronary artery bypass grafting (CABG) in the period 01/2000–07/2019. After surgery, patients received either SAPT (n = 153), or DAPT (n = 200) for six months then lifelong SAPT. Endpoints included early, late survival, and freedom from major-adverse-cardiac and cerebrovascular events (MACCE), which were defined as incidence of stroke, myocardial infarction, need for coronary intervention (PCI or CABG) or death for any cause. *Results*—Patients’ mean age was 67 ± 9.3 years; they were predominantly male 88.1%. Both DAPT- and SAPT-groups had the same extent of CAD (mean SYNTAX-Score-II: 34.1 ± 11.6 vs. 34.4 ± 17.2, *p* = 0.91). Postoperatively, no difference between DAPT- and SAPT-groups was reported in the incidence of low-cardiac-output syndrome (5% vs. 9.8%, *p* = 0.16), revision for bleeding (5% vs. 6.5% *p* = 0.64), 30-day mortality (4.5% vs. 5.2%, *p* = 0.8) or MACCE (7.5% vs. 11.8%, *p* = 0.19). Imaging follow-up reported significantly higher CEA and total grafts patency (90% vs. 81.5% and 95% vs. 81%, *p* = 0.017) in DAPT patients. Late outcomes within 97.4 ± 67.4 months show lower incidence of overall mortality (19 vs. 51%, *p* < 0.001) and MACCE (24.5 vs. 58.2%, *p* < 0.001) in the DAPT patients when compared with SAPT patients. *Conclusions*—Coronary endarterectomy allows revascularization in end-stage CAD when the myocardium is still viable. The use of dual APT after CEA for at least six months seems to improve mid-to-long-term patency rates and survival, and reduced the incidence of major adverse cardiac and cerebrovascular events.

## 1. Introduction

In the late 1950s, coronary endarterectomy (CEA) was introduced to allow the revascularization of severely diffused coronary artery disease (CAD) [1,2]. Since then, different techniques with controversial results have been reported, in which some outcomes were good [3,4] and others were negative [5,6]. CEA can be performed via either an open [7,8] or closed [9,10] technique. Similar to percutaneous coronary intervention (PCI), after CEA the injured remnants of the vessel’s media could initiate fast neo intimal tissue ingrowth. According to ESC/EACTS- and ACC/AHA-guidelines, “the use of dual antiplatelet therapy (DAPT) could protect against fast intimal ingrowth after injuring procedures by PCI” [11,12]. Moreover, the 2017 EACTs guidelines on perioperative medication in cardiac surgery recommended DAPT to be considered (IIb) in coronary artery bypass grafting (CABG) patients with higher ischemic risk (e.g., coronary endarterectomy or off-pump surgery) [13]. However, the role of antiplatelet therapy after CEA remains uncertain. We aimed in this study to review outcomes of patients undergoing CEA within CABG surgery who received either single antiplatelet therapy (SAPT) or DAPT after surgery and during follow-up.

## 2. Patients and Methods

### 2.1. Patient Population

A review board approval according to the University Hospital Ethics Committee (Ref# 19-8871-BO) was obtained to perform this retrospective observational study. Between 01/2000 and 07/2019, a total of 475 consecutive patients presenting with diffused CAD undergoing CEA within CABG surgery were evaluated. After screening, patients who underwent concomitant procedures were excluded, allowing a cohort of 353 patients undergoing isolated CABG surgery to be evaluated in this study. Patients’ perioperative data were collected in our institutional database and extracted for evaluation. All available preoperative angiographies were investigated to classify the extent of coronary artery disease according to the current SYNTAX score definitions.

Survival follow-up was achieved via reviewing all medical and civil office records. Postoperative imaging was realized for patients presenting either with anginal symptoms or for follow-up reasons. Clinical follow-up was accomplished via an active clinical interview using a standardized questionnaire (Supplementary Materials). Follow-up was complete through July 2021 reaching a mean time of 97.4 ± 67.4 (range of 12–228) months.

### 2.2. Surgery

**Figure 1:**
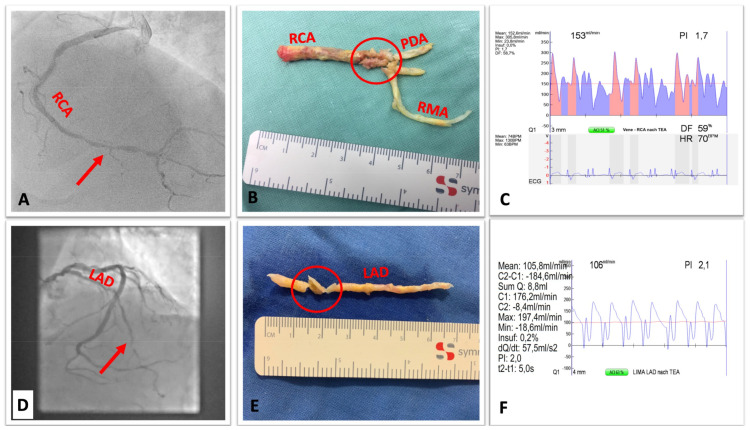
Various atheromatous cylinders extracted from different coronary-territories. Legend: The red arrow and the red circle refers to the site of incision, (**A**) angiography showing diffusely atherosclerotic RCA, (**B**) shows extracted cylinder from the main RCA and its trifurcation, (**C**) Pot-operative TTFM and PI within SVG→RCA bypass after CEA. (**D**) Angiography showing subtotal LAD occlusion, (**E**) shows the extracted atheroma from the LAD, (**F**) Postoperative TTFM and PI of the LIMA→LAD bypass after CEA. RCA = right coronary artery, PDA = posterior descending artery, RMA = right marginal artery, TTFM = transit time flow measurement, PI = pulsatility index, LAD = left anterior descending artery, SVG = saphenous vein grafting, LIMA = left internal mammary artery.

All surgeries were performed on-pump using cardiopulmonary bypass (CPB), under arrested heart after administration of crystalloid cardioplegia. Coronary endarterectomy was performed in the case of total or sub-total occlusion (i.e., if the vessel’s lumen < 1.25 mm). The criteria for performing CEA was the vessel´s outer diameter and viable surrounding myocardium worth to be preserved. Viable myocardium for all the three coronary territories was identified by performing preoperative cardiac magnetic resonance imaging when the ventricular function was known to be impaired and patient was presented for elective procedure [14], otherwise dobutamine stress echocardiography became a routine perioperative investigation to identify biventricular function and myocardial viability not only for the CEA territory but also for the non-CEA territories [15]. The applied CEA-technique was a modified closed-traction technique and consisted of five steps as earlier reported from our group [16]. A proper removal of the atheroma was considered when a smoothly tapered cylinder is extracted (Figure 1), otherwise distal second incision and subsequent anastomosis was performed to guarantee adequate revascularization of the peripheral segments. After anastomosis was undertaken with the chosen graft, transit time flow measurement (TTFM; MediStim, Oslo, Norway) [17] was applied to control all grafts’ function (Figure 1).

### 2.3. Postoperative Management

**Figure 2:**
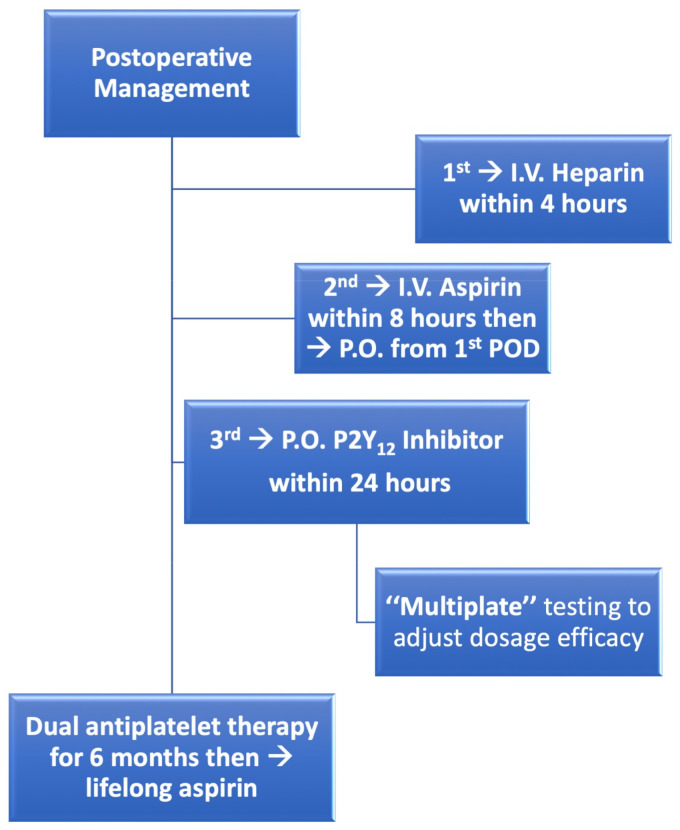
Postoperative management regime for patients undergoing coronary endarterectomy.

Since 2003, the usage of P2Y12 inhibitor using clopidogrel^®^ has been indicated and recommended in our institution in patients who were less or non-responding to acetylsalicylic acid (ASA, aspirin^®^) and sometimes as an adjuvant therapy in which CEA was performed in more than one coronary territory, this usage, however, was only implemented in sporadic cases and not as a routine. Later, the wide acceptance of adjuvant P2Y12 to ASA in patients undergoing coronary artery revascularization either via percutaneous intervention [18], or even after contrary artery bypass surgery [19], allowed us to change our regimen in patients having severely diffused CAD undergoing CEA within CABG surgery, where the application of P2Y12-inhibitor within 24 h and for 6 months achieved more adoption in our institution. Realizing that the addition of P2Y12-inhibitor did not increase the number of postoperative bleeding events, DAPT therapy using ASA and P2Y12 for six months became a routine regimen since 2008 according to our hospital guidelines. Figure 2 describes our postoperative management regimen for patients undergoing CABG and CEA from 2008 onwards. This consists of heparinization within the first four postoperative hours, followed by applying intravenous ASA within 8 h followed with oral ASA and P2Y12 inhibitor (without loading dose) from the first postoperative day and for six months. Thereafter patients were recommended to have lifelong ASA. Simultaneously, multiple platelet function analyzer (Multiplate^®^, Dynabyte GmbH, Munich, Germany) was used to adjust dosage efficacy and detect non-responses to the given ASA or P2Y12-inhibitor [20].

### 2.4. Endpoints

Study endpoints included early and long-term survival and freedom from major adverse cardiac and cerebrovascular events (MACCE). Graft failure is represented with incidence of postoperative myocardial infarction, graft occlusion demonstrated by imaging or cardiac-related death. LCOS was defined as perioperative need for intra-aortic balloon pump (IABP) or extra-corporeal membrane oxygenator (ECMO) support. A cerebrovascular event was defined with new incidence of stroke. MACCE included incidence of overall mortality, myocardial infarction, stroke or need for re-vascularization (stenting or re-CABG) as defined in the SYNTAX trial.

### 2.5. Statistical Analysis

**Figure 3:**
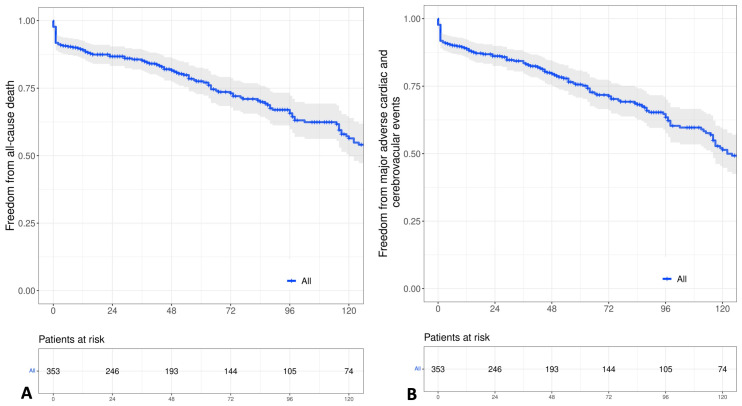
Kaplan-Meier curves showing long-term results regarding: (**A**) Overall estimated survival. (**B**) Overall freedom from MACCE.

**Figure 4:**
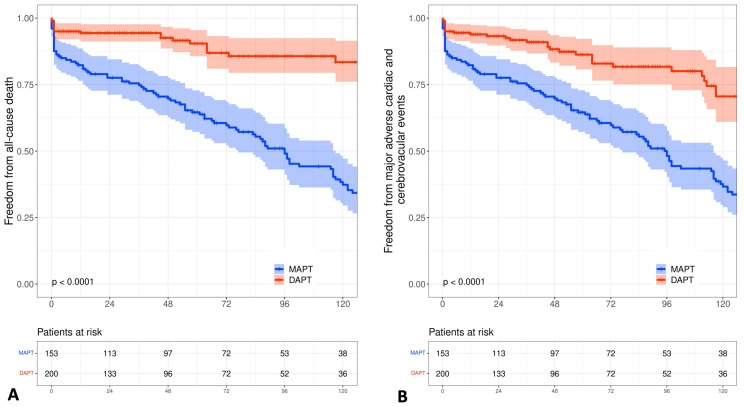
Kaplan-Meier curves illustrating difference between patients received dual-APT (DAPT) and single- or mono-APT (S/MAPT) groups regarding: (**A**) Estimated survival. (**B**) Freedom from MACCE.

Statistics were performed using the SPSS-software (version 22.0. IBM Corp., Armonk, NY, USA). Continuous data were expressed as mean ± standard deviation (SD) or median with interquartile ranges (IQR) (25th–75th percentiles) and compared between groups using the unpaired Student’s t-test or Mann Whitney U test when appropriate. Categorical data were expressed as frequencies and percentages and compared between groups using Chi-Square (χ2) test or Fisher’s exact test when appropriate. Reported *p*-values were two-sided and a value of *p* < 0.05 was considered statistically significant. Kaplan-Meier curves were generated to estimate total freedom from all-cause mortality or major adverse cardiac and cerebrovascular events and then in both groups (Figure 3 and Figure 4), where log-rank test was used to evaluate difference between both groups.

## 3. Results

### 3.1. Patients’ Demographics

Baseline characteristics are summarized in Table 1 reporting the evaluation of 353 consecutive patients who underwent CEA within isolated CABG. Mean age was 67 ± 9.3 years and most of the patients were male (311, 88.1%). Most patients (93.5%) had three-vessel disease. Mean SYNTAX-SCORE-II was 34.2 ± 19.4. Patients in the DAPT group were significantly older (68.7 ± 9.5 vs. 65.7 ± 8.6, *p* = 0.017) and there were more males (93 % vs. 81.7%, *p* = 0.004) than the SAPT group. More patients in the SAPT group presented with CCS class III-IV (42.5% vs. 35.5%, *p* < 0.0001)and NYHA class III-IV (51% vs. 15.5%, *p* < 0.0001) than patients in the DAPT group.

### 3.2. Early Outcomes

Table 2 summarizes intraoperative outcomes. All patients were operated under cardiopulmonary bypass with a mean cross-clamp time of 85 ± 19.4 min. A mean of 4.3 ± 1 grafts per patient were applied with a mean of 1.23 ± 0.5 CEAs, making a total of 435 CEAs. Sixty-eight (19.3%) of the patients required more than one CEA. Indication for CEA was either totally occluded (96, 27.2%), sub-totally occluded (249, 70.5%) coronaries or both (8, 2.3%). CEA was performed in the LAD- (191, 43.9%), RCA- (175, 40.2%), and/or LCX-territory (69, 15.9%). Grafts used after CEA were either arterial (145, 27.2%) or venous (249, 70.5%) or both (35, 9.9%). Transit time blood flow measurement over the CEA grafts after discontinuation of cardiopulmonary bypass (CPB) was 64 (43–94) mL/minute, and pulsatility index was 2.1 (1.6–2.9). Postoperatively patients received either single-antiplatelet-therapy (SAPT = 153) or dual-antiplatelet-therapy (DAPT = 200). Early postoperative outcomes are reported in Table 3. No significant difference between DAPT and SAPT groups was found in regard to incidence of LCOS (5% vs. 9.8%, *p* = 0.16), myocardial infarction in (1.5% vs. 3.9%, *p* = 0.183), stroke (1.5% vs. 3.3%, *p* = 0.3), revision for bleeding (5% vs. 6.5%, *p* = 0.64), ICU-Stay (2 (1–3) vs. 1.5 (1–3) days, *p* = 0.806) or operative mortality (4.5% vs. 5.2%, *p* = 0.8), respectively.

### 3.3. Late Outcomes

Survival follow-up was complete for 98.6% of patients (five patients were lost during follow-up). Long-term outcomes demonstrated a significant lower incidence of overall mortality (19% vs. 51%, *p* < 0.0001) and MACCE (24.5% vs. 58.2%, *p* < 0.0001) in the DAPT versus SAPT group, respectively, as reported in Table 4 and in Kaplan Meier curves for the estimation of survival and freedom from MACCE (Figure 4A,B). Table 5 summarizes detailed clinical and imaging follow-up results. Clinical follow-up for survivals was completed in 90% of the patients where 36 patients did not fill out the questionnaire. The clinical status of the surviving patients shows that most of the patients (89.3%) were completely independent on help and presented with NYHA I-II (90.8%). A significant difference between patients receiving dual- versus single-APT was observed in regard to the incidence of angina (5.6% vs. 17%, *p* = 0.02), myocardial infarction (2.1% vs. 13.2%, *p* = 0.004), percutaneous (but no surgical) revascularization (4.2% vs. 17%, *p* = 0.011). Imaging follow-up was present in 48.5% (95/196) of the surviving patients. Imaging was done using either conventional coronary angiography (53/95) or coronary computed-tomographic angiography (42/95). The indication for imaging was either angina (56/95) or for research reasons (39/95). Total grafts and CEA grafts patency rates were significantly higher in patients receiving DAPT in comparison to those receiving SAPT (95% vs. 81% and 90% vs. 81.5%, *p* = 0.017, respectively). Finally, from total survivors, eleven patients developed stroke, five patients underwent cardiac surgery other than CABG and thirteen patients required pacemaker implantation during follow-up.

## 4. Discussion

The main findings of this study are: 1. CEA can achieve myocardial revascularization in patients presented with end-stage coronary artery disease. 2. DAPT using ASA and P2Y12 for 6 months after CEA seems to protect against fast neo intimal ingrowth and prevent graft failure. 3. Imaging outcomes were only available in 48.5% of the patients due to patients’ refusal and financial causes. 4. Short-term results are acceptable and good mid- to long-term survival outcomes were observed.

Historically, CEA has been introduced as an option to allow revascularization in patients having severe and diffuse coronary artery diseases [1,2,21]. During surgery, CEA involves the removal of the atherosclerotic plaque “cylinder” which results in removing the media and intima of the coronary vessel. The remnants’ surface quickly proliferate and develop a fibrin platelet thrombosis which becomes organized fibrous tissue and may result in significant stenosis and early graft failure [22,23]. Moreover, according to the ESC/EACTS and ACC/AHA guidelines recommending DAPT after PCI for at least six months [11,12] to protect stenting against neo intimal tissue ingrowth. The current hypothesis suggests that the use of dual antiplatelet therapy after CEA could protect the injured remnants of the vessel’s media against fast neo intimal tissue ingrowth, which is in accordance with the 2017 EACTS guideline on perioperative medication in cardiac surgery recommending DAPT patients with higher ischemic risk (e.g., coronary endarterectomy) [13]. Similarly, in a large retrospective analysis testing the use of DAPT compared to monotherapy with ASA after CABG, there was a significant reduction in in-hospital mortality in the DAPT group (0.95% vs. 1.78%, *p* = 0.048) [24]. In another meta-analysis of randomized controlled trials, the use of DAPT seems to reduce mortality to 50% for patients with acute coronary syndrome undergoing CABG [25]. In the current study, we do observe a discrete reduction in 30-day and cardiac-related mortality in patients receiving DAPT. This reduction reaches statistical significance in later survival outcomes (Figure 4).

Imaging after coronary surgery is considered the best test of postoperative graft function. However, it was only available in 48.5% of survivals (95/196), mainly due to patients’ refusal and financial issues, although proposed by us to all patients. Other outcomes such as low-output syndrome, myocardial infarction and cardiac-cause mortality would indirectly indicate early graft failure. In this study, low-output syndrome was reported in 7.1% of patients, 2.5% of patients had perioperative myocardial infarction. Thirty-day mortality was reported in 4.8% of patients, with cardiac-related death in 3.1% of patients, one-year and overall mortality in 8.8% and 32.9% of patients. Notably, 29.5% of patients underwent urgent/emergent surgery, which might impact early perioperative results, especially mortality. These results are comparable to earlier published data [4,26]. Imaging follow-up at a mean of 58.1 ± 57.6 months demonstrated significantly higher patency rates of all grafts including those endarterectomized in patients, who received DAPT, compared to those receiving only aspirin (95% vs. 81% and 90% vs. 81.5%, *p* = 0.017, respectively). Graft patency is usually influenced by different factors such as previous myocardial infarction in the territory of the graft, poor run-off into the periphery of the endarterectomized vessel, incomplete removal of the atheromatous plaques or residual intimal flap may result in early occlusion as reported by the Cooley group [27], the type of conduit used, as well as intraoperative graft flow. In this study, coronary bypass flow was significantly higher in DAPT patients than in SAPT patients (65 (45–90) vs. 57 (35–80) mL/minute, *p* = 0.028), as proven by TTFM measurements after discontinuation of CPB, even though this finding was only observed retrospectively, graft flow should be considered for long-term graft patency evaluation. As expected, most of the occluded grafts were venous (37 out of 45 of the total grafts and 15 out of 17 of the CEA grafts), which corresponds to previous reports observing better arterial graft patency in comparison to the venous ones [28,29]. A probable explanation for graft patency might be the role of early DAPT therapy starting at the first postoperative day using ASA + P2Y12-inhibitor for six months.

To compensate for the limitation of deficient imaging follow-up, an intimate active clinical follow-up through a personal and/or phone-call interview with a standardized questionnaire (appendix) was established with around 90% completeness. At the last follow-up, patients in the dual-APT group reported significantly lower mortality (19% vs. 51%, *p* < 0.0001), less incidence of angina (5.6% vs. 17%, *p* = 0.02), myocardial infarction (2.1% vs. 13.2%, *p* = 0.004), percutaneous revascularization (4.2% vs. 17%, *p* = 0.011), and overall incidence of MACCE (24.5% vs. 58.2%, *p* < 0.0001) compared to those treated with SAPT. Moreover, the clinical status of the surviving patients reported that most of the patients were completely independent on help (89.3%) and having no or mild limitation of physical activity according to the NYHA classification (NYHA I-II in 90.8% of patients).

Generally, outcomes after coronary endarterectomy are different due to its complexity, with reported results varying from being acceptable to very bad. In the study by the Cooley group examining more than 30,000 patients undergoing either CABG adjunctive with CEA compared with CABG alone, CEA + CABG significantly increased operative mortality (4.4% vs. 2.6%, *p* = 0.01) and postoperative MI (5.4% vs. 2.6%, *p* = 0.01) but had comparable long-term outcomes [27]. Similarly, in the recent analysis of the society of thoracic surgeons’ adult cardiac surgery database (STS ACSD) by Kelly and colleagues, CEA + CABG was associated with increased rates of operative mortality (3.2% vs. 1.7%; *p* < 0.0001; OR, 1.81; 95% CI, 1.63–2.01) and postoperative MI (6.8% vs. 3.9%; *p* < 0.0001; OR, 1.80; 95% CI, 1.68–1.93). However, long-term survival was similar after the first year, and readmission for MI was comparable after the third year [30]. In contrast, results from Myers and associates reported an operative mortality of 3 to 4.1% and an incidence of perioperative myocardial infarction in 4 to 4.1% depending on the technique of CEA, either using vein patch or direct LAD grafting, respectively [26]. More recently, the group of Takanashi et al. reported a 30-day mortality of 1.1% and a perioperative myocardial infarction rate of 9% in patients after CEA of the LAD and left internal thoracic artery (LITA) grafting [4]. Although coronary artery endarterectomy remains rare in current practice, it has continued to be used as a bail-out option in patients presented with end-stage diffuse coronary artery disease undergoing coronary artery bypass grafting with acceptable short- to long-term survival outcomes.

## 5. Study Limitations

One limitation of the current study is that both groups were not matched in regard to their preoperative characteristics which might impact the results. The study’s basis as a retrospective observational single centre is another limitation. Those possibilities of selection bias call for randomized controlled trials to investigate the value of our findings. Postoperative imaging was only available in 48.5% of the patients due to patients’ refusal or financial reasons. However, survival follow-up was 98.5% complete and strict clinical follow-up was achieved in about 90% of patients.

## 6. Conclusions

Coronary endarterectomy enables surgical revascularization in patients with severe and diffuse coronary artery disease with satisfactory short- and long-term outcomes in spite of its complexity. Supported by concomitant dual antiplatelet therapy, mid- to long-term outcomes seem to be significantly improved. Thus, the presented study could initiate a prospective multicentre randomized controlled trial for the evaluation of the reported promising results.

## Figures and Tables

**Table 1:** Baseline demographic characteristics.

	All Patients (*n* = 353)	Single-APT (*n* = 153)	Dual-APT (*n* = 200)	*p*-Value
***Demographics***				
Age, years	67 ± 9.3	65.7 ± 8.6	68.7 ± 9.5	0.017
Gender, males	311 (88.1)	125 (81.7)	186 (93)	0.004
Body mass index, kg/m^2^	27.4 ± 4.0	27.7 ± 4.3	27.1 ± 3.8	0.196
***Risk factors and comorbidities***				
Diabetes mellitus	133 (37.7)	55 (35.9)	78 (39)	0.38
Systemic hypertension	310 (87.8)	134 (87.6)	175 (87.5)	1.0
Active smoker	67 (19)	26 (17)	41 (20.5)	0.306
Hypercholesterinaemia	225 (63.7)	96 (62.7)	129 (64.5)	0.824
COPD	39 (11.0)	20 (13.1)	19 (9.5)	0.307
Peripheral vascular disease	59 (16.7)	29 (18.9)	30 (15)	0.388
Central vascular disease	62 (17.6)	23 (15)	39 (19.5)	0.578
Preoperative dialysis	6 (1.7)	2 (1.3)	4 (2)	0.702
Previous cerebrovascular event	27 (7.6)	10 (6.5)	17 (8.5)	0.547
Previous myocardial infarction	140 (39.7)	60 (39.2)	80 (40)	0.913
Previous CABG	20 (5.7)	11 (7.2)	9 (4.5)	0.245
Non-elective surgery	104 (29.5)	54 (35.3)	50 (25)	0.035
CCS III-IV	136 (38.5)	65 (42.5)	71 (35.5)	<0.0001
NYHA III-IV	109 (30.9)	78 (51)	31 (15.5)	<0.0001
***Extent of coronary artery disease***				0.126
Three-vessel disease	330 (93.5)	142 (92.8)	188 (94)	
Two-vessel disease	20 (5.7)	8 (5.2)	12 (6)	
One-vessel disease	3 (0.8)	3 (2)	0	
***SYNTAX-scores***				
SYNTAX-SCORE I	27.4 ± 9.3	28.2 ± 9.6	26.8 ± 9.0	0.174
SYNTAX-SCORE II	34.2 ± 19.4	34.4 ± 27.17	34.1 ± 11.6	0.91
***Left ventricular function***				0.81
EF > 50%	219 (62)	98 (64.0)	121 (60.5)	
EF = 30–50%	111 (31.4)	46 (30.1)	65 (32.5)	
EF < 30%	23 (6.5)	9 (5.9)	14 (7)	
***Risk scores***				
Logistic EuroSCORE I	2.8 (1.5–4.5)	3.7 (1.8–8.2)	2.9 (1.5–4.6)	0.863
EuroSCORE II	1.3 (1–2.1)	1.6 (0.94–1.8)	1.3 (0.88–2.3)	0.722

Data are presented as mean ± SD, Median (IQR) or number and (%). COPD = Chronic obstructive pulmonary diseases, CCS = Canadian Cardiovascular Society grading of angina pectoris, NYHA = New York Heart Association, CABG= coronary artery bypass grafting, EF = ejection fraction.

**Table 2:** Intraoperative Outcomes.

	All Patients (*n* = 353)	Single-APT (*n* = 153)	Dual-APT (*n* = 200)	*p*-Value
Aortic cross-clamp time, minutes	85 ± 19.4	85.4 ± 18.4	84.9 ± 20.2	0.81
CBP-time = bypass time, minutes	121.6 ± 31.5	121.9 ± 30.3	121.4 ± 32.5	0.872
Total number of grafts	1534	641	893	--
Mean number of grafts/patient	4.3 ± 1.0	4.2 ± 1.1	4.5 ± 1.0	0.17
Total number of CEA-grafts	435	171	264	--
Mean number of CEA-graft/patient	1.23 ± 0.51	1.1 ± 0.4	1.3 ± 0.6	0.1
***Number of CEA-grafts in each patient***				0.001
One-CEA	285 (80.7)	137 (89.5)	148 (74)	
Two-CEAs	56 (15.9)	14 (9.2)	42 (21)	
Three-CEAs	10 (2.8)	2 (1.3)	8 (4)	
Four-CEAs	2 (0.6)	0	2 (1)	
***Indication of CEA***				0.006
Totally occluded vessels	96 (27.2)	53 (34.6)	43 (21.5)	
Sub-totally occluded vessels	249 (70.5)	99 (64.7)	150 (75)	
both	8(2.3)	1(0.7)	7(3.5)	
***Graft used after CEA***				0.23
Arterial graft	116 (32.9)	49 (32)	67 (33.5)	
Venous graft	202 (57.2)	96 (62.7)	106 (53)	
Arterial and Venous graft	35 (9.9)	8 (5.2)	27 (13.5)	
***TTFM of CEA graft after CPB***				
TTFM	64 (43–94)	57 (35–80)	65 (45–90)	0.028
Pulsatility index	2.1 (1.6–2.9)	2 (2–3.5)	2.1 (1.5–2.8)	0.849
**CEA was done at**	***n* = 435**	***n* = 171**	***n* = 264**	
LAD-Territory	191 (43.9)	72 (42.1)	119 (45.1)	0.133
RCA-Territory	175 (40.2)	61 (35.7)	114 (43.2)	0.013
LCX-Territory	69 (15.9)	38 (22.2)	31 (11.7)	0.029

Data are presented as mean ± SD or number and (%). CEA = coronary endarterectomy, TTFM = transit time flow measurement, CPB = cardiopulmonary bypass, LAD = left anterior descending artery, LCX = left circumflex artery, RCA = right coronary artery.

**Table 3:** Postoperative outcomes.

	All Patients (*n* = 353)	Single-APT (*n* = 153)	Dual-APT (*n* = 200)	*p*-Value
Low cardiac output syndrome	25 (7.1)	15 (9.8)	10 (5)	0.16
Need for IABP	18 (5.1)	10 (6.5)	8 (4)	
Need for ECMO	7 (2)	5 (3.3)	2 (1)	
Myocardial infarction	9 (2.5)	6 (3.9)	3 (1.5)	0.183
Stroke	8 (2.3)	5 (3.3)	3 (1.5)	0.3
Revision for bleeding	20 (5.7)	10 (6.5)	10 (5)	0.64
Packed red cell transfusion	660 (600–800)	750 (75–502.5)	600 (0–1200)	0.827
Temporary dialysis	33 (9.3)	16 (10.5)	17 (8.5)	0.58
Respiratory complications	42 (11.9)	17(11.1)	25 (12.5)	0.94
Need for re-intubation	18 (5.1)	7 (4.6)	11 (5.5)	
Need for tracheostomy	24 (6.8)	10 (6.5)	14 (7)	
Deep wound infection	13 (3.7)	3 (2)	10 (5)	0.162
ICU-stay, days	1.6 (1–3.5)	1.5 (1–3)	2 (1–3)	0.806
Hospital stay, days	12.1 ± 10.1	11.8 ± 10.7	12.4 ± 9.6	0.59
Operative mortality				
30-day mortality	17 (4.8)	8 (5.2)	9 (4.5)	0.8
Cardiac-related mortality	11 (3.1)	6 (3.9)	5 (2.5)	
Operative MACCE	33 (9.3)	18 (11.8)	15 (7.5)	0.19

Data are presented as mean ± SD or number and (%). IAPB = intra-aortic balloon pump, ECMO = extracorporeal membrane oxygenation, ICU = intensive care unit.

**Table 4:** Long-term outcomes.

	All Patients (*n* = 353)	Single-APT (*n* = 153)	Dual-APT (*n* = 200)	*p*-Value
Lost during follow-up	5 (1.4)	3 (2)	2 (1)	
Mortality at one year	31 (8.8)	16 (10.6)	15 (7.5)	0.347
Mortality at five years	94 (26.6)	58 (37.9)	36 (18)	<0.0001
Overall mortality at last follow-up	116 (32.9)	78 (51)	38 (19)	<0.0001
Survivors at last follow-up	232 (65.7)	72 (47)	160 (80)	<0.0001
Patients did not fill out the questionnaire	36 (10.2)	19 (12.4)	17 (9)	
Overall MACCE at last follow-up	138 (39.1)	89 (58.2)	49 (24.5)	<0.0001

Data are presented as number and (%) MACCE = major adverse cardiac and cerebrovascular events.

**Table 5:** Clinical and imaging outcomes.

Questionnaire Validation	Total Number (*n* = 196)	Single-APT (*n* = 53)	Dual-APT (*n* = 143)	*p*-Value
***Clinical follow-up***				
Completely independent	175 (89.3)	42 (79.2)	133 (93)	0.012
Depends on help for daily routine	21 (10.7)	11 (20.8)	10 (7)	0.012
NYHA-class I-II	178 (90.8)	43 (81.1)	135 (94.4)	0.018
NYHA-class III-IV	18 (9.2)	10 (18.9)	8 (5.6)	0.018
Stroke	11 (5.6)	5 (9.4)	6 (4.2)	0.172
Angina pectoris	17 (8.7)	9 (17)	8 (5.6)	0.02
Myocardial infarction	10 (5.1)	7 (13.2)	3 (2.1)	0.004
PCI/Stenting	15 (7.7)	9 (17)	6 (4.2)	0.011
Re-CABG	0			
Other cardiac surgery	5 (2.6)	4 (7.5)	1 (0.7)	0.027
Pacemaker implantation	13 (6.6)	6 (11.3)	7 (4.9)	0.037
***Imaging follow-up***				
Imaging	95 (48.5)	39 (73.6)	56 (39)	<0.0001
Coronary catheter	53	29	24	
Computed tomography	42	10	32	
Indication for Imaging	95 (48.5)	39 (73.6)	56 (39)	<0.0001
Symptoms	56	24	32	
Research	39	15	24	
Graft patency				
CEA-graft patency	107/124 (86.3)	44/54 (81.5)	63/70 (90)	0.017
Total graft patency	378/423 (89.4)	136/168 (81)	242/255 (95)	0.017

Data are presented as number and (%). NYHA = New York Heart Association functional classification, PCI = percutaneous coronary intervention, CABG= coronary artery bypass grafting, CEA= coronary endarterectomy.

## Data Availability

Not applicable.

## Supplementary Materials

The following supporting information can be downloaded at: https://www.mdpi.com/article/10.3390/jcdd10030112/s1, Our follow-up standardized questionnaire in English translation.
