# Supplementary material for: Role of Antiplatelet Therapy in Patients with Severe Coronary Artery Disease Undergoing Coronary Artery Endarterectomy within Coronary Artery Bypass Surgery
Source: J Cardiovasc Dev Dis. 2023 Mar 7;10(3):112. doi: 10.3390/jcdd10030112 (PMC10051999; doi:10.3390/jcdd10030112)
Supplement: Supplementary file 1 [file jcdd-10-00112-s001.zip › jcdd-2216829-supplementary.pdf]

University Hospital Essen	Postoperative follow-up ''Questionnaire''	WHGZ

**Name of the Patient:**	**Date of birth:**
**Which operation:**	**Date of surgery:**

We have the following couple of questions regarding your health status after surgery:

1. **How you manage your daily tasks?** Independent/ dependent on help
2. **Did you have a stroke since surgery?** No / Yes → Date……………………………………
   Do you have a neurological deficit? No / Yes → which one?…………………………
3. **Do you have any lung disease?** No / Yes → which one?…………………………
4. **Do you have any limitation of physical activity (NYHA classification)?** No / Yes→Class........
   Class I: No limitation of physical activity.
   Class II: Slight limitation of physical activity.
   Class III: Marked limitation of physical activity.
   Class IV: Unable to carry out any physical activity without discomfort.
5. **Did you have any chest pain radiating to the neck/jaw, to the left/right shoulder/arm or upper belly since surgery?** No / Yes → Since when?…………………………
6. **Did you have myocardial infarction after surgery?** No / Yes → Date…………………………..
7. **Did you have coronary angiography after surgery?** No / Yes → Date…………………………..
8. **Did you have stent-Implantation after surgery?** No / Yes → Date……………………………….
9. **Did you undergo a new coronary artery bypass surgery?** No / Yes → Date……………………………
10. **Did you have any valvular infection after surgery?** No / Yes → Since when?…………………..
    Which valve was / is affected ?...

**Filled by:**<br><br>**Follow-up date:**		**Approved and released by:**<br>*Associate Professor Dr. S.-E. Shehada*<br>"Consultant cardiac surgeon"

11. **Did you have any valvular regurgitation after surgery?** No / Yes → Since when?…………

    Which valve was / is affected ?...

12. **Did you undergo another cardiac surgery?** No / Yes → Date………………………

    Which operation you had ?...

13. **Do you have heart arrhythmias?**

    Atrial fibrillation No / Yes → Since when?…………

    Another arrhythmia No / Yes → Since when?…………

    Pacemaker implantation No / Yes → Date………………………

14. **Did you have kidney disease or insufficieny after surgery?** No / Yes

    Are you on dialysis? No / Yes → Since when?…………

15. **Do you have peripheral or central vascular disease?** No / Yes → which one?………..

    Did you need surgery or stent implantation No / Yes → Date……………………

16. **Did you acquire another disease or undergo any other surgery?** No / Yes → Date…………………

    Which operation you had ?..

17. **When was the last echocardiographic control?** Date…………………………………….

    Can we have a copy of the echocardiographic report? No / Yes

    Do you have an appointment for the next examination? No / Yes → Date…………………

18. **What is the name of your cardiologist / GP ?** Name…………………., City……………………………….

19. **In case we still have questions, is it okay to call you ?** No / Yes, Phone number.:.....................

Remarks:...

....

Patient's signature……….……………………………………….

We thank you for your patience and effort!

**Filled by:**<br><br>**Follow-up date:**		**Approved and released by:**<br>*Associate Professor Dr. S.-E. Shehada*<br>"Consultant cardiac surgeon"
